# Rabies and rabies prophylaxis in Egypt: a nationwide cross-sectional study that calls for action

**DOI:** 10.1186/s12889-026-29562-9

**Published:** 2026-10-15

**Authors:** Mohamed Basyouni Helal, Mohamed Ahmed Saeda, Ahmed Gamal Elshaar, Mohamed Elsayed Zayed, Ali M. Abdelaziz, Omnia M. Mostafa, Enas Sherif Adwy, Ahmed Barakat, Ibrahim Mohammed Abdelkader, Basma Amir Eldawody, Noor Maged Badrawy Ahmed, Mohanned Noah, Ashraf Aboismail, Abdelrahman Mohamed Mahmoud, Abdallah R. Allam

**Affiliations:** 1 Faculty of Medicine, Menoufia University, Yassin Abdelghaffar Street from Gamal Abdelnaser Street, Shebin Al-Kom, Menoufia 32511 Egypt; 2 Faculty of Medicine, Alexandria University, Alexandria, Egypt; 3 Faculty of Medicine, South Valley University, Qena, Egypt; 4 Faculty of Medicine, Benha University, Qalubia, Egypt; 5 Faculty of Medicine, Assiut University, Assiut, Egypt; 6 Faculty of Medicine, Suez University, Suez, Egypt; 7 Faculty of Medicine, Mansoura University, Mansoura, Egypt; 8 Faculty of Medicine, Ain Shams University, Cairo, Egypt; 9 Faculty of Medicine, Cairo University, Cairo, Egypt; 10 Department of Tropical Medicine, Faculty of Medicine, Alexandria University, Alexandria, Egypt

**Keywords:** Rabies, Rabies vaccine, Practices, Knowledge, Attitudes, Egypt

## Abstract

**Background:**

Rabies is a fatal but preventable viral disease that poses a global health threat, especially in developing countries. Despite the high incidence of animal bites reported annually in Egypt, limited data exist regarding the knowledge, attitudes, and practices (KAP) of the general population toward rabies and rabies prophylaxis.

**Methods:**

We conducted a nationwide cross-sectional study using a structured interview-based survey targeting individuals aged 18 years or older who attended outpatient clinics at nine university hospitals across Egypt. The survey assessed participants’ KAP toward rabies and rabies prophylaxis and identified barriers to prophylaxis uptake.

**Results:**

Among 1,777 participants who responded to the survey, only 42.8% (*n* = 760) had good knowledge about rabies and rabies prophylaxis, while the majority, 88.5% (*n* = 1,572), exhibited a positive attitude toward rabies prophylaxis. Knowledge was higher among those who reported awareness of rabies campaigns in their geographic areas (69.1% vs. 40.5%, *p* < 0.001) and those who had previously been bitten or attacked by animals (56.1% vs. 41.3%, *p* < 0.001). Overall, 9.7% (*n* = 173) of participants reported a history of animal bites or attacks; however, 18.5% (*n* = 32) of these participants did not receive post-exposure prophylaxis (PEP), and 17% (*n* = 24) did not complete the vaccine schedule. Pre-exposure prophylaxis (PrEP) uptake was low, with only 4.3% (*n* = 23) of high-risk individuals receiving it. Key barriers to rabies prophylaxis included lack of knowledge about the rabies vaccine, reported by 82.4% of participants, lack of knowledge about rabies, reported by 81.7%, and inadequate hospital facilities, reported by 43.1%.

**Conclusions:**

Despite a generally positive attitude toward rabies prophylaxis, knowledge and adherence to prophylactic measures, including pre-exposure and post-exposure prophylaxis, remain relatively inadequate in Egypt. Public health interventions, such as educational campaigns and improved accessibility to vaccination services, are needed to address these gaps and mitigate the public health burden of rabies.

**Supplementary Information:**

The online version contains supplementary material available at https://doi.org/10.1186/s12889-026-29562-9.

## Introduction

Rabies remains one of the most serious and feared threats to public health in the 21st century. Rabies has been associated with high fatality rates, resulting in 59,000 deaths worldwide every year, with 36% of cases occurring in Africa [1]. The majority of deaths occur in rural communities in developing countries in Africa and Asia [2], with a large economic burden estimated at USD 8.6 billion [1].

The disease is caused by the rabies virus, which belongs to the Lyssavirus genus [3]. Rabies is transmitted to humans through the saliva of infected animals, with dogs being responsible for 99% of human cases [4]. Once clinical symptoms appear, rabies is virtually always fatal [3, 5].

Fortunately, rabies deaths are preventable through prompt and appropriate post-exposure prophylaxis (PEP), while pre-exposure prophylaxis (PrEP) is recommended for individuals at high risk of exposure [6]. However, in developing countries, challenges exist regarding adherence to PEP; for example, a study in Senegal found that only 50% of patients completed the full regimen [7].

In Egypt, the Ministry of Health and Population reported 200,000 animal bites each year [8], making the need for proper knowledge and practices regarding rabies prophylaxis crucial. However, data regarding knowledge and adherence to rabies prophylaxis are insufficient. While a survey assessing knowledge and practice toward rabies has been conducted, the data were limited to a single center and a small sample, which is not fully representative of the general population [9]. In addition, data regarding attitudes and practices remain limited.

Therefore, the present study aimed to evaluate knowledge and attitudes toward rabies and rabies prophylaxis among the general population in Egypt. Additionally, we aimed to explore practices and barriers to rabies prophylaxis to provide insights for improving rabies prevention and control strategies in Egypt.

## Methods

### Study design and setting

**Fig. 1:**
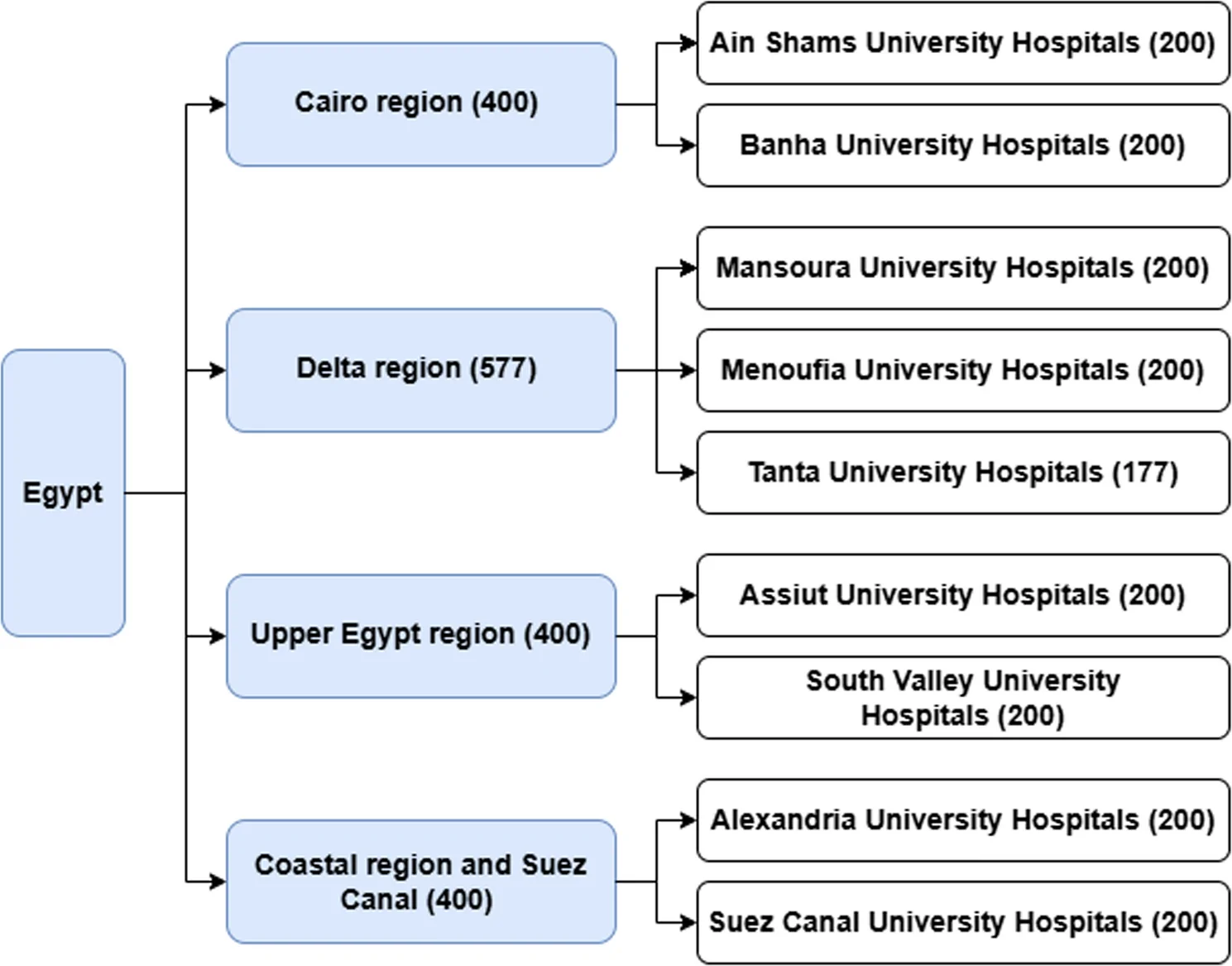
Study centers overview

We conducted a nationwide questionnaire-based cross-sectional study in Egypt to assess rabies prophylaxis practices from the perspective of both the general and high-risk populations. Participants were recruited from outpatient clinics at nine university hospitals, geographically distributed across Egypt (the Cairo region, the Coastal and Suez Canal regions, the Delta region, and Upper Egypt). A complete list of the included study centers is provided in Fig. 1.

### Study population and sample

The study included participants aged 18 years and older who attended outpatient clinics or waiting rooms at the selected study centers. Students in the medical field, healthcare workers, and individuals under the age of 18 were excluded.

### Sampling and sample size calculation

A convenience sampling method was used to recruit eligible study participants. The sample size was calculated using the online Raosoft sample size calculator [10], with the following parameters: a 95% confidence level, an expected frequency of 50%, and an acceptable margin of error of 2.5%. The anticipated sample size was 1,428, which was later increased to 1,777 during data collection to compensate for any potential incomplete responses or data loss.

### Questionnaire development and validation

A structured questionnaire was developed specifically for this study to assess rabies prophylaxis practices in Egypt from the perspectives of both the general population and high-risk groups. The primary objective was to evaluate knowledge, attitudes, barriers, and practices related to rabies prophylaxis.

Following a comprehensive review of the literature, the study team created an initial draft of the questionnaire. This draft was reviewed and discussed by two experts in the field to ensure its relevance and clarity. A pilot test was conducted with 20 participants to evaluate the clarity, accuracy, and relevance of each question, as well as the duration required for completion. Based on the feedback from the pilot study, the questionnaire was refined and finalized. Participants included in the pilot study were excluded from the final analysis. The finalized questionnaire included multiple sections: sociodemographic data, history and awareness of rabies, knowledge of rabies and its prophylaxis, attitudes toward rabies and its prophylaxis, barriers to rabies prophylaxis, and practices related to rabies prophylaxis.

The sociodemographic section collected data on participants’ gender, age, occupation, highest level of education, income, region, and residency. The history section examined participants’ prior awareness of rabies, personal experiences with animal attacks, and their connections to individuals who had been affected by rabies. The knowledge section contained 11 closed-ended questions: 7 focused on knowledge about rabies and 4 on knowledge of rabies prophylaxis. The attitude section included 7 questions aimed at gauging participants’ views on the risk and fatality of rabies, as well as their willingness to undergo both post-exposure prophylaxis and pre-exposure prophylaxis. The barriers section explored factors that could prevent individuals from seeking rabies prophylaxis, such as lack of knowledge, fear, financial burdens, or previous negative experiences with healthcare. Finally, the practices section focused on participants’ actual behaviors regarding rabies prophylaxis, distinguishing between those who had experienced animal bites and received post-exposure treatment and those who belonged to high-risk groups recommended for pre-exposure prophylaxis.

The questionnaire was originally developed in English and then translated into Arabic by a language specialist to ensure that it was understandable to the local population. A full version of the questionnaire can be found in Supplementary file 1.

### Data collection

A team of well-trained data collectors was assigned to each university to conduct data collection. Participants were approached in their respective university outpatient clinics and waiting rooms. Data collectors received standardized instructions regarding participant eligibility, informed consent, questionnaire administration, and consistent recording of responses. The same finalized Arabic questionnaire and structured interview approach were used across all nine study centers to ensure consistency in data collection.

### Ethical considerations

The study was conducted according to the principles of the Declaration of Helsinki (1964, last revised in 2013) [11]. Participation was voluntary, and all participants provided informed consent before data collection. All participants also received detailed explanations about the aim, objectives, and methodology of the study before completing the questionnaire. Participants’ anonymity and confidentiality were ensured throughout the study, including during data collection and analysis. Ethical approval was obtained from the Ethical Committee for Scientific Research at the Faculty of Medicine, Alexandria University.

### Statistical analysis

The data were analyzed using IBM SPSS Statistics, version 28.0, for Windows (Armonk, NY: IBM Corp.). Frequencies and percentages were used to describe categorical variables and baseline demographic characteristics. The normality of continuous variables was assessed using the Shapiro-Wilk and Kolmogorov-Smirnov tests. The median and interquartile range (IQR) were used to describe non-normally distributed continuous variables. Pearson’s chi-square test, Fisher’s exact test, and the Fisher-Freeman-Halton exact test were used to explore associations between categorical variables. A p-value < 0.05 was considered statistically significant.

For the knowledge questions, answers were coded as 1 for correct and 0 for incorrect, yielding a score range of 0–11 per participant. If a question had multiple correct answers (e.g., animals that transmit rabies), the response was considered correct if the participant selected at least half of the correct options. Participants with a knowledge score of ≥ 6 were categorized as having good knowledge, whereas those with scores of 0–5 were categorized as having poor knowledge. For the attitude section, responses to closed-ended questions (e.g., recognizing rabies as a health risk or supporting prophylaxis) were coded as 1 for positive attitudes and 0 for negative attitudes. For questions using a 1–10 scale, scores of ≥ 6 were coded as 1 and scores < 6 as 0, yielding a total attitude score ranging from 0 to 7. Participants with an attitude score of ≥ 4 were categorized as having a positive attitude, whereas those with scores of 0–3 were categorized as having a negative attitude. Descriptive analyses included frequencies and percentages of correct answers and positive attitudes, as well as the median and interquartile range (IQR) for both knowledge and attitude scores.

## Results

### Demographic characteristics of the participants

A total of 1,777 participants from nine Egyptian centers were included in the study, with a median age of 35 years (IQR = 23). Of these, 52% (*n* = 924) were females and 48% (*n* = 853) were males. About 75.7% (*n* = 1,345) of our sample had secondary education or higher, and 52.4% (*n* = 931) were from rural areas. Almost 70% (*n* = 1,244) of the participants worked in occupations not considered vulnerable to rabies exposure, and 50.7% (*n* = 901) reported having sufficient income. A significant majority of participants, 79.1% (*n* = 1,405), had heard about rabies, with family and friends being the primary source of information for 62.6% (*n* = 1,113). Meanwhile, only 5% (*n* = 89) had previously discussed rabies with a physician. Awareness of rabies vaccination campaigns was reported by only 7.8% (*n* = 139) of respondents. Overall, 50.8% (*n* = 903) of participants knew someone who had been bitten or attacked by an animal, while only 7.7% (*n* = 136) knew someone who had been diagnosed with or had died from rabies. Additionally, 9.7% (*n* = 173) of participants had personally been bitten or attacked by an animal **(**Table 1**).**

**Table 1:** Sociodemographic characteristics and rabies-related history of the study participants (*N* = 1,777)

Variable	*n* (%)
Age, Median (IQR)	35 (23)
Age category	
− 18–35	905 (50.9%)
− 36–60	707 (39.8%)
− > 60	165 (9.3%)
Gender	
− Male	853 (48%)
− Female	924 (52%)
Region	
− Cairo region	400 (22.5%)
− Coastal region and Suez Canal	400 (22.5%)
− Delta Region	577 (32.5%)
− Upper Egypt	400 (22.5%)
Residency	
− Rural	931 (52.4%)
− Urban	846 (47.6%)
Occupation	
− Vulnerable	533 (30%)
− Not vulnerable	1244 (70%)
Highest level of education	
− Non-educated	188 (10.6%)
− Primary School	244 (13.7%)
− Secondary School	607 (34.2%)
− College	702 (39.5%)
− Postgraduate Studies	36 (2%)
Income	
− Not enough	840 (47.3%)
− Enough	901 (50.7%)
− Enough and sometime saving	36 (2%)
Heard about rabies before	
− Yes	1405 (79.1%)
− No	372 (20.9%)
Sources of knowledge about rabies	
− Radio/Television	255 (14.4%)
− Social media	340 (19.1%)
− School	149 (8.4%)
− Family/Friend	1113 (62.6%)
Discussion with a physician about rabies	
− Yes	89 (5%)
− No	1688 (95%)
Awareness or vaccination campaigns are held for rabies in your geographical area.	
− Yes	139 (7.8%)
− No	1638 (92.2%)
Been bitten or attacked by an animal before	
− Yes	173 (9.7%)
− No	1604 (90.3%)
Know anyone been bitten or attacked by an animal before	
− Yes	903 (50.8%)
− No	874 (49.2%)
Know anyone diagnosed or died from rabies before	
− Yes	136 (7.7%)
− No	1641 (92.3%)

### Knowledge

The median knowledge score among respondents was 5 (IQR: 3) on a 0–11 scale. Overall, 42.8% (*n* = 760) of participants demonstrated good knowledge about rabies, with scores of 6 or higher. Only 25.9% (*n* = 461) of participants correctly identified rabies as a viral disease, while 13.1% (*n* = 232) could name the animals that transmit rabies. A significant majority, 88.4% (*n* = 1,570), were aware of the mode of transmission of rabies. Only a minority of participants knew the signs of rabies in animals and humans, 20.9% (*n* = 371) and 16.4% (*n* = 291), respectively. About 81.2% (*n* = 1,443) believed that a person attacked by a rabid animal could recover with treatment. However, only 18.4% (*n* = 327) were aware that rabies is untreatable once clinical signs appear. About half of our sample, 50.1% (*n* = 890), knew the appropriate first aid measures after an animal attack, 73.9% (*n* = 1,314) knew that a rabies vaccine existed, and a majority, 81.9% (*n* = 1,455), knew when rabies post-exposure prophylaxis should be initiated. About 47.8% (*n* = 849) of participants were knowledgeable about the appropriate candidates for pre-exposure prophylaxis **(**Table 2**).**

**Table 2:** Knowledge and attitudes regarding rabies and rabies prophylaxis among study participants (*N* = 1,777)

Questions	*N* (%) of correct answer*	Questions	*N* (%) of positive attitude
Participants who knew what rabies is.	461 (25.9%)	Participants thought that rabies is a risk to human health.	1620 (91.2%)
Participants who knew the animals that transmit rabies.	232 (13.1%)	Rate the level of danger from rabies on a scale of 1 to 10 (1 = very mild, 10 = very severe), Median (IQR)	9 (4)
Participants who knew the mode of transmission of rabies.	1570 (88.4%)	Participants thought that rabies is a fatal disease.	1457 (82%)
Participants who knew signs of rabies in animals.	371 (20.9%)	On a scale of 10, how do you support undergoing rabies post-exposure prophylaxis? (1 = strongly disagree, 10 = strongly agree), Median (IQR)	10 (2)
Participants who knew signs of rabies in human beings.	291 (16.4%)	On a scale of 10, how do you recommend undergoing rabies pre-exposure prophylaxis for the vulnerable group? (As 1 = strongly disagree and 10 = strongly agree), Median (IQR)	9 (4)
Participants who thought a man was attacked by a rabid animal can recover with treatment.	1443 (81.2%)	Participants thought that injecting a rabies vaccine as soon as possible after being bitten by suspected rabid animals is a must.	1597 (89.9%)
Participants who knew if rabies can’t be treated after the onset of clinical signs.	327 (18.4%)	Participants thought that completing the full course of vaccination after being bitten by a suspected rabid animal is a must.	1579 (88.9%)
Participants who knew the first aid after an animal attack.	890 (50.1%)	Attitude score (total 7 points), Median (IQR)	7 (1)
Participants who know that there is a vaccine for rabies.	1314 (73.9%)	The overall attitude category − Positive attitude (4–7 points) − Negative attitude (0–3 points)	1572 (88.5%) 205 (11.5%)
Participants who knew when rabies post-exposure prophylaxis should be taken.	1455 (81.9%)		
Participants who knew who should take the pre-exposure prophylaxis of rabies.	849 (47.8%)		
Knowledge score, Median (IQR)	5 (3)		
The overall knowledge category − Good knowledge (6–11 points) − Poor knowledge (0–5 points)	760 (42.8%) 1017 (57.2%)		

Data is presented as number (percentage) or median (IQR). * In questions that have multiple correct answers, a response is considered correct if the participant selects at least half or more of the correct options

Our study found that good knowledge was higher among participants from urban areas compared with rural areas (50.7% vs. 35.6%, *p* < 0.001), among male participants compared with females (48.9% vs. 37.1%, *p* < 0.001), among participants with occupations vulnerable to rabies (66.2% vs. 32.7%, *p* < 0.001), and among participants with sufficient or higher income compared with those with low income (55.7% and 77.8% vs. 27.4%, respectively; *p* < 0.001). Good knowledge was also higher among participants who reported awareness of rabies vaccination campaigns (69.1% vs. 40.5%, *p* < 0.001), those who had previously heard about rabies (49.8% vs. 16.1%, *p* < 0.001), those with a history of animal attacks (56.1% vs. 41.3%, *p* < 0.001), those who knew someone who had been bitten or attacked by an animal (51.2% vs. 34.1%, *p* < 0.001), and those who knew someone who had been diagnosed with or had died from rabies (66.2% vs. 40.8%, *p* < 0.001).

A significant association was found between geographic region and good knowledge, with the highest proportion observed in Cairo (52.3%; *p* < 0.001). Educational level was also significantly associated with good knowledge, with higher proportions among college graduates and participants with postgraduate education (50.3% and 63.9%, respectively; *p* < 0.001). The source of rabies information was also significantly associated with good knowledge; participants who obtained information from school had a higher proportion of good knowledge than those who obtained information from radio/television (76.5% vs. 40.4%, *p* < 0.001) **(**Table 3**).**

**Table 3:** Bivariate associations of participant characteristics with knowledge and attitudes regarding rabies and rabies prophylaxis

Variable	Good Knowledge (*n* = 760)	Poor Knowledge (*n* = 1017)	*P*-value	Positive Attitude (*n* = 1572)	Negative Attitude (*n* = 205)	*P*-value
Age − 18–35	406 (44.9%)	499 (55.1%)	0.131	844 (93.3%)	61 (6.7%)	**< 0.001**
− 36–60	292 (41.3%)	415 (58.7%)		619 (87.6%)	88 (12.4%)	
− > 60	62 (37.6%)	103 (62.4%)		109 (66.1%)	56 (33.9%)	
Gender − Male	417 (48.9%)	436 (51.1%)	**< 0.001**	753 (88.3%)	100 (11.7%)	0.813
− Female	343 (37.1%)	581 (62.9%)		819 (88.6%)	105 (11.4%)	
Region − Cairo region	209 (52.3%)	191 (47.8%)	**< 0.001**	395 (98.8%)	5 (1.2%)	**< 0.001**
− Coastal region and Suez Canal	177 (44.2%)	223 (55.8%)		246 (61.5%)	154 (38.5%)	
− Delta Region	253 (43.8%)	324 (56.2%)		576 (99.8%)	1 (0.2%)	
− Upper Egypt	121 (30.2%)	279 (69.8%)		355 (88.8%)	45 (11.2%)	
Residency − Rural	331 (35.6%)	600 (64.4%)	**< 0.001**	837 (89.9%)	94 (10.1%)	**0.046**
− Urban	429 (50.7%)	417 (49.3%)		735 (86.9%)	111 (13.1%)	
Highest level of education − Non-educated	75 (39.9%)	113 (60.1%)	**< 0.001**	157 (83.5%)	31 (16.5%)	**< 0.001**
− Primary School	103 (42.2%)	141 (57.8%)		217 (88.9%)	27 (11.1%)	
− Secondary School	206 (33.9%)	401 (66.1%)		498 (82%)	109 (18%)	
− College	353 (50.3%)	349 (49.7%)		664 (94.6%)	38 (5.4%)	
− Postgraduate Studies	23 (63.9%)	13 (36.1%)		36 (100%)	0 (0)	
Occupation − Vulnerable	353 (66.2%)	180 (33.8%)	**< 0.001**	529 (99.2%)	4 (0.8%)	**< 0.001**
− Not vulnerable	407 (32.7%)	837 (67.3%)		1043 (83.8%)	201 (16.2%)	
Income − Not enough	230 (27.4%)	610 (72.6%)	**< 0.001**	675 (80.4%)	165 (19.6%)	**< 0.001**
− Enough	502 (55.7%)	399 (44.3%)		861 (95.6%)	40 (4.4%)	
− Enough and sometime saving	28 (77.8%)	8 (22.2%)		36 (100%)	0 (0)	
Heard about rabies before − Yes	700 (49.8%)	705 (50.2%)	**< 0.001**	1304 (92.8%)	101 (7.2%)	**< 0.001**
− No	60 (16.1%)	312 (83.9%)		268 (72%)	104 (28%)	
Sources of knowledge about rabies						
− Radio/Television	103 (40.4%)	152 (59.6%)	**< 0.001**	197 (77.3%)	58 (22.7%)	**< 0.001**
− Social media	164 (48.2%)	176 (51.8%)		309 (90.9%)	31 (9.1%)	
− School	114 (76.5%)	35 (23.5%)		148 (99.3%)	1 (0.7%)	
− Family/Friend	571 (51.3%)	542 (48.7%)		1084 (97.4%)	29 (2.6%)	
Discussion with a physician about rabies − Yes	41 (46.1%)	48 (53.9%)	0.519	77 (86.5%)	12 (13.5%)	0.555
− No	719 (42.6%)	969 (57.4%)		1495 (88.6%)	193 (11.4%)	
Awareness or vaccination campaigns are held for rabies in your geographical area. − Yes	96 (69.1%)	43 (30.9%)	**< 0.001**	138 (99.3%)	1 (0.7%)	**< 0.001**
− No	664 (40.5%)	974 (59.5%)		1434 (87.5%)	204 (12.5%)	
Been bitten or attacked by an animal before − Yes	97 (56.1%)	76 (43.9%)	**< 0.001**	163 (94.2%)	10 (5.8%)	**0.013**
− No	663 (41.3%)	941 (58.7%)		1409 (87.8%)	195 (12.2%)	
Know anyone been bitten or attacked by an animal before − Yes	462 (51.2%)	441 (48.8%)	**< 0.001**	830 (91.9%)	73 (8.1%)	**< 0.001**
− No	298 (34.1%)	576 (65.9%)		742 (84.9%)	132 (15.1%)	
Know anyone diagnosed or died from rabies before − Yes	90 (66.2%)	46 (33.8%)	**< 0.001**	135 (99.3%)	1 (0.7%)	**< 0.001**
− No	670 (40.8%)	971 (59.2%)		1437 (87.6%)	204 (12.4%)	
Knowledge level of rabies − Good knowledge				756 (99.5%)	4 (0.5%)	**< 0.001**
− Poor knowledge				816 (80.2%)	201 (19.8%)	

### Attitude

The median attitude score among respondents was 7 (IQR: 1) on a 0–7 scale. Overall, 88.5% (*n* = 1,572) exhibited a positive attitude toward rabies and rabies prophylaxis, with scores of 4 or higher. Most participants, 91.2% (*n* = 1,620), considered rabies a risk to human health, and 82% (*n* = 1,457) considered it a fatal disease **(**Table 2**).**

A significantly higher frequency of positive attitudes was observed among rural residents compared with urban residents (89.9% vs. 86.9%, *p* = 0.046) and among participants aged 18–35 years compared with those aged 36–60 years and > 60 years (93.3% vs. 87.6% and 66.1%, respectively; *p* < 0.001). Participants from the Delta region and Cairo also demonstrated a higher frequency of positive attitudes (99.8% and 98.8%, respectively; *p* < 0.001). Educational level was significantly associated with attitude, with a higher frequency of positive attitudes among college graduates and those with postgraduate education (94.6% and 100%, respectively; *p* < 0.001). A significantly higher frequency of positive attitudes was also observed among participants with sufficient or higher income compared with those with low income (95.6% and 100% vs. 80.4%, respectively; *p* < 0.001), those with occupations vulnerable to rabies (99.2% vs. 83.8%, *p* < 0.001), participants who knew someone who had previously been bitten or attacked by an animal (91.9% vs. 84.9%, *p* < 0.001) or knew someone who had been diagnosed with or died from rabies (99.3% vs. 87.6%, *p* < 0.001), participants who had previously been bitten or attacked by an animal (94.2% vs. 87.8%, *p* = 0.013), participants who had previously heard about rabies (92.8% vs. 72%, *p* < 0.001), and participants who reported awareness of rabies vaccination campaigns (99.3% vs. 87.5%, *p* < 0.001).

Participants with good knowledge also had a higher frequency of positive attitudes (99.5% vs. 80.2%, *p* < 0.001). Similarly, participants whose source of rabies information was school had a higher frequency of positive attitudes than those whose source was radio/television (99.3% vs. 77.3%, *p* < 0.001). In contrast, gender (*p* = 0.813) and discussions with physicians (*p* = 0.555) were not significantly associated with attitude **(**Table 3**).**

### Post-exposure prophylaxis

Most participants in our study, 90.3% (*n* = 1,604), had no history of animal attacks. A subgroup analysis was conducted among the 9.7% (*n* = 173) of participants with a history of animal bites or attacks. Of these, 18.5% (*n* = 32) did not receive post-exposure prophylaxis. Among those who received PEP, 6.4% (*n* = 9) initiated the first dose more than 24 h after exposure, while 0.7% (*n* = 1) initiated vaccination after 1–2 weeks. In addition, 17% (*n* = 24) did not complete the full vaccine schedule **(**Table 4**).** We investigated the associations between participant characteristics and receipt of the post-exposure rabies vaccine among these 173 participants. Significant associations were found for age (*p* = 0.014) and region (*p* = 0.002), with the highest uptake observed in Upper Egypt (98%). Educational level was also significantly associated with vaccine uptake (*p* = 0.006). Having previously heard about rabies (*p* = 0.020), social media as a source of rabies information (*p* = 0.035), discussion with a physician about rabies (*p* = 0.015), and awareness of rabies vaccination campaigns (*p* = 0.045) were significantly associated with vaccine uptake. Knowledge level was significantly associated with post-exposure vaccine uptake (*p* = 0.005). Gender (*p* = 0.828), residency (*p* = 0.166), occupation (*p* = 0.527), income (*p* = 0.605), and attitude toward rabies (*p* = 0.396) were not significantly associated with vaccine uptake **(**Table 5**).**

**Table 4:** Rabies prophylaxis practices among study participants

Post-exposure prophylaxis			
	Total		*n* (%)
Did you undergo an attack or be bitten by an animal?	1777	Yes	173 (9.7%)
		No	1604 (90.3%)
If you have been attacked, did you take the rabies post-exposure vaccine from the nearest hospital?	173	Yes	141 (81.5%)
		No	32 (18.5%)
When did you start your first dose?	141	Within the first 24 h of the time of being bitten	131 (92.9%)
		After more than 24 h of biting exposure time	9 (6.4%)
		After 1–2 weeks	1 (0.7%)
Did you complete the full vaccine schedule (total 5 doses)?	141	Yes	117 (83%)
		No	24 (17%)
Pre-exposure prophylaxis			
Are you one of the vulnerable populations for rabies?	533	Animal control officers	4 (0.8%)
		Farmers or people who live in rural areas.	319 (59.8%)
		People who have pets at home	82 (15.4%)
		People who live in context with stray dogs and cats.	120 (22.5%)
		Travelers to rabies-endemic area	4 (0.8%)
		Wildlife workers	2 (0.4%)
Did you think it’s a must to take the pre-exposure prophylaxis?	533	Yes	208 (39%)
		No	325 (61%)
Did you take the rabies pre-exposure vaccine from the nearest hospital?	533	Yes	23 (4.3%)
		No	510 (95.7%)
Did you complete the vaccine schedule (total 3 doses)?	23	Yes	18 (78.3%)
		No	5 (21.7%)

**Table 5:** Bivariate associations of participant characteristics with receipt of post-exposure prophylaxis among participants with a history of animal bites or attacks

Variable	Took the post-exposure vaccine after an animal attack. (*n* = 141)	Didn’t take the post-exposure vaccine after an animal attack. (*n* = 32)	*P*-value
Age − 18–35	77 (89.5%)	9 (10.5%)	0.014
− 36–60	53 (71.6%)	21 (28.4%)	
− > 60	11 (84.6%)	2 (15.4%)	
Gender − Male	91 (82%)	20 (18%)	0.828
− Female	50 (80.6%)	12 (19.4%)	
Region − Cairo region	38 (73.1%)	14 (26.9%)	**0.002**
− Coastal region and Suez Canal	13 (65%)	7 (35%)	
− Delta Region	42 (80.8%)	10 (19.2%)	
− Upper Egypt	48 (98%)	1 (2%)	
Residency − Rural	72 (85.7%)	12 (14.3%)	0.166
− Urban	69 (77.5%)	20 (22.5%)	
Highest level of education − Non-educated	15 (57.7%)	11 (42.3%)	**0.006** ^**a**^
− Primary School	19 (76%)	6 (24%)	
− Secondary School	44 (86.3%)	7 (13.7%)	
− College	61 (89.7%)	7 (10.3%)	
− Postgraduate Studies	2 (66.7%)	1 (33.3%)	
Occupation − Vulnerable	84 (80%)	21 (20%)	0.527
− Not vulnerable	57 (83.8%)	11 (16.2%)	
Income − Not enough	70 (83.3%)	14 (16.7%)	0.605^a^
− Enough	66 (78.6%)	18 (21.4%)	
− Enough and sometime saving	5 (100%)	0 (0)	
Heard about rabies before − Yes	136 (83.4%)	27 (16.6%)	0.020*
− No	5 (50.0%)	5 (50.0%)	
Sources of knowledge about rabies			
− Radio/Television	19 (82.6%)	4 (17.4%)	> 0.999*
− Social media	32 (94.1%)	2 (5.9%)	**0.035**
− School	12 (100%)	0 (0)	0.126*
− Family/Friend	107 (82.3%)	23 (17.7%)	0.635
Discussion with a physician about rabies − Yes	21 (100%)	0 (0)	0.015*
− No	120 (78.9%)	32 (21.1%)	
Awareness or vaccination campaigns are held for rabies in your geographical area. − Yes	17 (100%)	0 (0)	0.045*
− No	124 (79.5%)	32 (20.5%)	
Know anyone been bitten or attacked by an animal before − Yes	109 (79.6%)	28 (20.4%)	0.200
− No	32 (88.9%)	4 (11.1%)	
Know anyone diagnosed or died from rabies before − Yes	23 (85.2%)	4 (14.8%)	0.789*
− No	118 (80.8%)	28 (19.2%)	
Knowledge level of rabies − Good knowledge	72 (74.2%)	25 (25.8%)	**0.005**
− Poor knowledge	69 (90.8%)	7 (9.2%)	
Attitude towards rabies − Positive Attitude	134 (82.2%)	29 (17.8%)	0.396*
− Negative Attitude	7 (70%)	3 (30%)	

Data is presented as number (percentage). * Indicates the use of the Fisher Exact test. ^a^ Indicates the use of the Fisher-Freeman-Halton Exact Test

### Pre-exposure prophylaxis

Approximately one-third of the participants, 30.0% (*n* = 533), were considered a vulnerable population for rabies. Of these, 59.8% (*n* = 319) were farmers or individuals living in rural areas, 22.5% (*n* = 120) lived in environments with stray dogs and cats, and 15.4% (*n* = 82) had pets at home. Only 4.3% (*n* = 23) of participants received rabies pre-exposure prophylaxis, and 18 of them completed the full vaccination schedule **(**Table 4**).** We also investigated the associations between participant characteristics and pre-exposure rabies vaccine uptake among 533 vulnerable participants. Significant associations were found for region (*p* < 0.001), residency (*p* < 0.001), educational level (*p* < 0.001), specific sources of rabies information, including social media and school (*p* < 0.001 for both) and family/friends (*p* = 0.009), discussions with physicians about rabies (*p* < 0.001), awareness of vaccination campaigns (*p* < 0.001), and previous animal attacks (*p* < 0.001). No significant associations were found for age (*p* = 0.134), gender (*p* = 0.538), income (*p* = 0.155), previous awareness of rabies (*p* = 0.280), or attitude toward rabies (*p* > 0.999) **(**Table 6**).**

**Table 6:** Bivariate associations of participant characteristics with receipt of pre-exposure prophylaxis among participants considered vulnerable to rabies exposure

Variable	Took the pre-exposure vaccine. (*n* = 23)	Didn’t take the pre-exposure vaccine. (*n* = 510)	*P*-value
Age − 18–35	16 (6.1%)	246 (93.9%)	0.134
− 36–60	6 (2.6%)	222 (97.4%)	
− > 60	1 (2.3%)	42 (97.7%)	
Gender − Male	15 (4%)	363 (96%)	0.538
− Female	8 (5.2%)	147 (94.8%)	
Region − Cairo region	0 (0)	64 (100%)	**< 0.001** ^**a**^
− Coastal region and Suez Canal	5 (4%)	120 (96%)	
− Delta Region	6 (2%)	301 (98%)	
− Upper Egypt	12 (32.4%)	25 (67.6%)	
Residency − Rural	6 (1.6%)	375 (98.4%)	**< 0.001**
− Urban	17 (11.2%)	135 (88.8%)	
Highest level of education − Non-educated	1 (1.7%)	57 (98.3%)	**< 0.001** ^**a**^
− Primary School	3 (3.3%)	89 (96.7%)	
− Secondary School	3 (1.3%)	222 (98.7%)	
− College	16 (10.5%)	136 (89.5%)	
− Postgraduate Studies	0 (0)	6 (100%)	
Income − Not enough	4 (2.1%)	186 (97.9%)	0.155
− Enough	18 (5.4%)	313 (94.6%)	
− Enough and sometime saving	1 (8.3%)	11 (91.7%)	
Heard about rabies before − Yes	21 (4.8%)	413 (95.2%)	0.280*
− No	2 (2%)	97 (98%)	
Sources of knowledge about rabies			
− Radio/Television	3 (6.5%)	43 (93.5%)	0.438*
− Social media	13 (17.6%)	61 (82.4%)	< 0.001*
− School	7 (20.6%)	27 (79.4%)	< 0.001*
− Family/Friend	11 (2.9%)	372 (97.1%)	**0.009**
Discussion with a physician about rabies − Yes	8 (25%)	24 (75%)	< 0.001*
− No	15 (3%)	486 (97%)	
Awareness or vaccination campaigns are held for rabies in your geographical area. − Yes	9 (17.3%)	43 (82.7%)	< 0.001*
− No	14 (2.9%)	467 (97.1%)	
Been bitten or attacked by an animal before − Yes	12 (11.4%)	93 (88.6%)	< 0.001*
− No	11 (2.6%)	417 (97.4%)	
Know anyone been bitten or attacked by an animal before − Yes	15 (4.7%)	304 (95.3%)	0.591
− No	8 (3.7%)	206 (96.3%)	
Know anyone diagnosed or died from rabies before − Yes	6 (8.8%)	62 (91.2%)	> 0.999*
− No	17 (3.7%)	448 (96.3%)	
Knowledge level of rabies − Good knowledge	11 (3.1%)	342 (96.9%)	0.056
− Poor knowledge	12 (6.7%)	168 (93.3%)	
Attitude towards rabies − Positive Attitude	23 (4.3%)	506 (95.7%)	> 0.999*
− Negative Attitude	0 (0)	4 (100%)	

Data presented as number (percentage) *Indicates the use of the Fisher Exact test. ^a^ Indicates the use of the Fisher-Freeman-Halton Exact Test

### Barriers

**Fig. 2:**
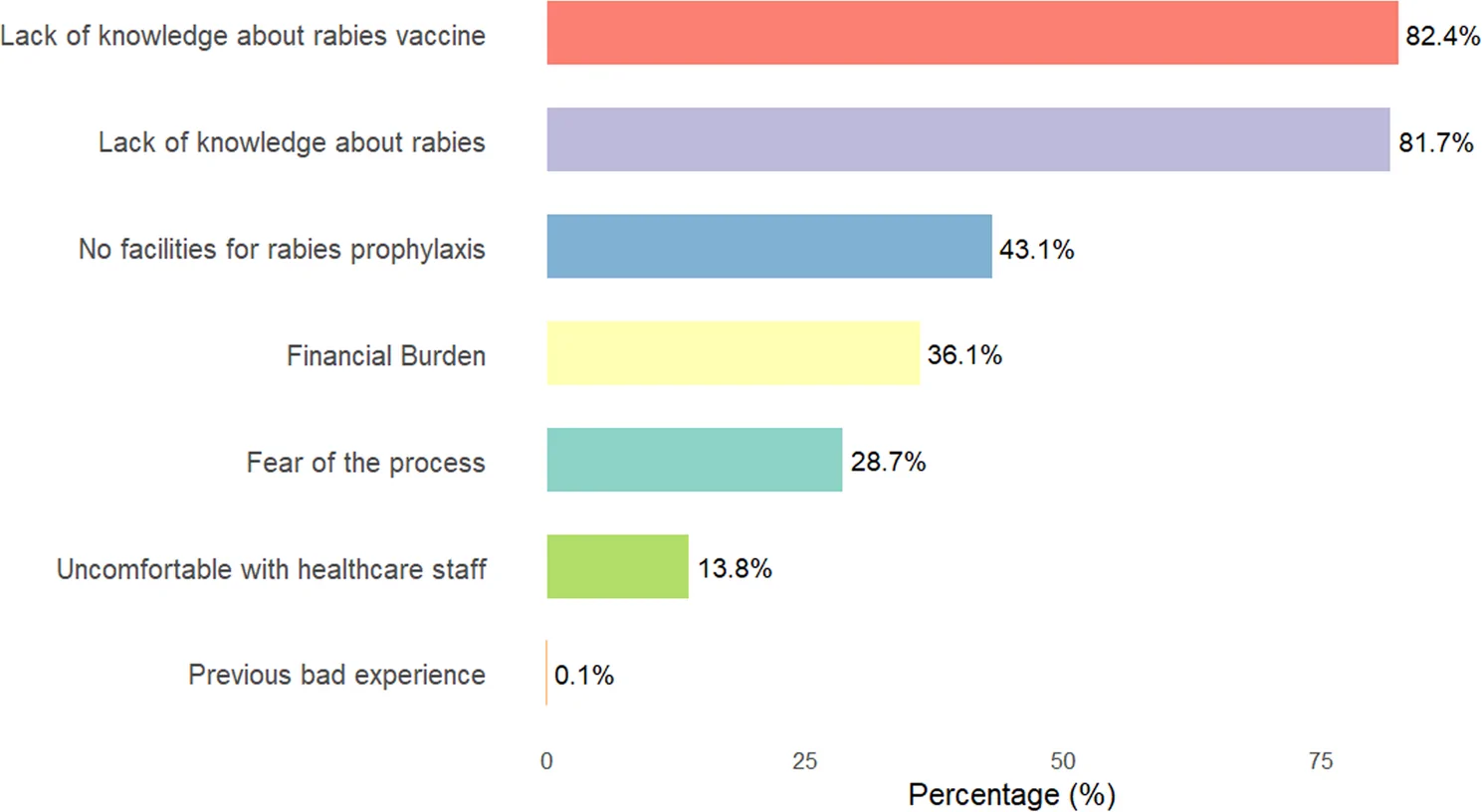
Barriers to rabies prophylaxis

Lack of knowledge about the rabies vaccine and rabies were the most frequently reported barriers to undergoing rabies prophylaxis, reported by 82.4% and 81.7% of participants, respectively. Other barriers included inadequate hospital facilities, reported by 43.1% of participants; financial burden, 36.1%; fear of the process, 28.7%; discomfort when dealing with healthcare staff, 13.8%; and previous negative experiences, 0.1% **(**Fig. 2**).** When asked to choose the single most important factor that would prevent them from undergoing rabies prophylaxis, 40.5% (*n* = 719) selected lack of knowledge about rabies as the most important barrier. This was followed by lack of knowledge about the vaccine, 23.4% (*n* = 415); lack of required facilities for rabies prophylaxis at local hospitals, 14.0% (*n* = 249); financial burden, 11.7% (*n* = 208); fear of the process, 8.0% (*n* = 142); and discomfort when dealing with healthcare staff, 1.6% (*n* = 29).

## Discussion

Despite being preventable through timely and appropriate vaccination and post-exposure prophylaxis, rabies poses a significant public health challenge in regions with inadequate healthcare infrastructure and low awareness levels, particularly in Asia and Africa [4]. Understanding the public’s knowledge about rabies is crucial for designing effective awareness campaigns, training healthcare workers, and improving preventive measures. In line with the WHO goal of eliminating deaths from dog-mediated rabies by 2030 [12], this study represents the first multicenter effort in Egypt to assess knowledge, attitudes, and practices (KAP) toward rabies, as well as barriers preventing access to proper care.

Our study revealed that less than half of the participants demonstrated good knowledge about rabies and rabies prophylaxis. However, more than 88% of participants reported positive attitudes toward rabies prevention. In addition, more than 18% of participants with a history of animal attacks did not receive post-exposure prophylaxis. Among vulnerable populations, only 23 participants received pre-exposure prophylaxis. Barriers to prophylaxis included mainly a lack of knowledge and other factors such as financial burden and insufficient hospital facilities.

The current study revealed that 42.8% of the participants were classified as knowledgeable about rabies, which is higher than findings from studies in Guatemala (4%) [13] and Tanzania (37%) [14], yet lower than the findings reported in Kenya (67%) [15], North India (57.9%) [16], and Ethiopia (49%) [17]. These variations could be attributed to differences in community knowledge, educational status, and access to information. Additionally, participants showed a good level of knowledge regarding the mode of rabies transmission. However, they were often unable to name the specific animals that transmit the disease, similar to findings in Guatemala [13] and Sri Lanka [18]. Only a minority of participants could identify the signs of rabies in animals and humans, contrary to the results from Bangladesh [19] and Tanzania [14], where knowledge levels were considerably higher.

Furthermore, geographic region was significantly associated with the level of rabies knowledge. This may be attributed to differences in the availability of vaccination and awareness programs across regions, particularly in rural areas, where dogs roam freely and are not routinely vaccinated, thus increasing the risk of transmission [20].

Good knowledge was also associated with awareness of vaccination campaigns, prior awareness of rabies, a history of animal attacks, and higher educational levels, notably among college graduates and those pursuing postgraduate degrees. Sources of rabies information, such as school, family, and friends, played an important role in providing information about the disease. These findings highlight important gaps in knowledge regarding the potential sources, prevention, and control of rabies, which may increase the population’s vulnerability to the disease.

In comparison to other studies, our findings revealed that 82% of participants recognized rabies as a fatal disease, which is consistent with two studies conducted in the Puducherry region in India, where recognition of rabies as a fatal disease ranged between 74.8% and 77.5% [21, 22]. The high proportion of participants who recognized rabies as fatal in our sample suggests that the public may be more receptive to vaccination programs, potentially improving their uptake and effectiveness.

Similar to previous studies, the study participants exhibited a positive attitude toward the rabies vaccine [23, 24]. In our study, 89.9% believed that after an animal bite, post-exposure prophylaxis should be received as soon as possible, and 88.9% believed that the PEP course should be completed. Such positive attitudes indicate that strengthening hospital-based PEP programs could help prevent rabies-related deaths. However, these attitudes should be converted into actual behavior through continuous public education emphasizing the importance of timely medical attention after any animal bite. Regular vaccination of pets and appropriate post-exposure care could substantially contribute to achieving national and international goals of eliminating dog-mediated human rabies deaths by 2030 [12].

Indiscriminate culling of dogs may undermine rabies-control efforts and can be counterproductive to vaccination programs. Mass canine vaccination remains the principal strategy for controlling dog-mediated rabies. Given the population’s positive attitude toward rabies prevention, national strategies should prioritize sustained animal vaccination programs. According to WHO guidance, vaccination coverage of at least 70% of the dog population should be targeted to effectively control canine rabies [25].

Income was found to be associated with attitudes toward rabies. Participants reporting insufficient income had a lower frequency of positive attitudes toward vaccination. This finding is similar to previous research showing that socioeconomic factors were associated with attitudes and practices related to rabies [24]. One possible explanation is that participants with higher socioeconomic status may have better access to health information and healthcare services, facilitating adherence to recommended management and vaccination. Participants in occupations vulnerable to rabies exposure also demonstrated a higher frequency of positive attitudes. This may reflect their greater knowledge of rabies due to their increased likelihood of exposure compared with individuals in non-vulnerable occupations.

Despite our participants’ positive attitudes toward PEP, 9.7% of participants in our study had previously been bitten or attacked by an animal, and 18.5% did not seek PEP. Our findings are in line with another study that found that 20% of subjects did not seek PEP following an animal bite [26]. This may partly reflect cultural influences on healthcare-seeking behavior, including seeking care from local healers. Delayed or absent access to appropriate PEP may increase the risk of rabies and its associated burden on the healthcare system. Therefore, improving the availability and accessibility of rabies prophylaxis in hospitals and healthcare facilities may help prevent rabies-related deaths and reduce the associated healthcare burden.

Moreover, 95.7% of the vulnerable group did not receive pre-exposure prophylaxis, and 61% of them did not believe that prophylaxis was necessary. This might be explained by restricted access to medical facilities, which may influence their knowledge and subsequent preventive behavior regarding rabies. Since participants with a college education had the highest uptake of pre-exposure prophylaxis, differences in educational level and health literacy may partly explain this finding.

Participants aged 36–60 years showed lower uptake of post-exposure prophylaxis than those aged > 60 years. Previous research has reported more appropriate first-aid practices among older individuals, including the use of soap and water or available antiseptic solutions following animal bites [27]. This may reflect greater health consciousness and a tendency to seek medical care promptly.

Raising public knowledge remains a cornerstone of rabies control [28]. Our findings demonstrated a strong association between knowledge and attitude, with participants who had good knowledge showing a higher frequency of positive attitudes. This is broadly consistent with Monje et al., who reported associations between knowledge, attitudes, and practices toward rabies prevention [29]. Primary care physicians should therefore play a central role in correcting misconceptions and promoting preventive and post-exposure care at the community level.

Our study identified several barriers to rabies prophylaxis. The predominant barriers were lack of knowledge about the rabies vaccine (82.4%) and rabies itself (81.7%), consistent with previous findings [30]. Another important barrier was inadequate hospital facilities (43.1%), which aligns with reports that restricted access to vaccines and trained staff can hinder timely PEP administration [31, 32]. Additional barriers included financial burden (36.1%), fear of the process (28.7%), discomfort with healthcare staff (13.8%), and previous negative experiences (0.1%). Addressing these issues requires a comprehensive approach, combining public education, healthcare system strengthening, improved access to prophylaxis, and better patient–provider communication.

## Conclusion

While most participants recognized rabies as a fatal and preventable disease, deficiencies persisted in specific knowledge areas, particularly regarding transmission routes, early signs, and appropriate prophylactic measures. Adherence to both pre- and post-exposure prophylaxis was suboptimal. Implementing structured national awareness programs could enhance public understanding and promote adherence to prophylaxis guidelines, ultimately contributing to Egypt’s efforts to eliminate dog-mediated human rabies deaths by 2030.

## Supplementary Information

Supplementary Material 1: Knowledge, attitudes, and practices toward rabies and rabies prophylaxis: a nationwide survey.

## Data Availability

The datasets analyzed during the current study are available from the corresponding author on reasonable request.

## Abbreviations

CI: Confidence interval
COVID-19: Coronavirus disease 2019
IQR: Interquartile range
KAP: Knowledge, attitudes, and practices
n: Number of participants
p: P-value
PEP: Post-exposure prophylaxis
PrEP: Pre-exposure prophylaxis
SPSS: Statistical package for the social sciences
STROBE: Strengthening the reporting of observational studies in epidemiology
USD: United States Dollar
WHO: World health organization
