# Supplementary material for: Rabies and rabies prophylaxis in Egypt: a nationwide cross-sectional study that calls for action
Source: BMC Public Health. 2026 Oct 15;26:2979. doi: 10.1186/s12889-026-29562-9 (PMC13670857; doi:10.1186/s12889-026-29562-9)
Supplement: Supplementary file 1 [file 12889_2026_29562_MOESM1_ESM.docx]

This is a 10-minute survey consisting of five main sections. Data are collected as part of a nationwide study to assess the knowledge, attitude towards rabies, and the current status of rabies prophylaxis practice in Egypt.

**PERSONAL DETAILS**

**Gender:**

Ο Male Ο Female

**Region**

**Age: “**Please write in English numbers”

**Occupation:** “In Arabic”

**Highest Level of Education:**

Ο Non-educated

Ο Primary School

Ο Secondary School

Ο College

Ο Postgraduate Studies

**Income:**

Ο Not enough

Ο Enough

Ο Enough and sometime saving

**Residency:**

Ο Urban Ο Rural

**HISTORY**

**Have you heard about rabies before?**

Ο Yes Ο No

**What are your sources of knowledge?** “You can choose more than one answer.”

Ο Radio/Television

Ο Social media

Ο School

Ο Family/Friend

Ο Previous discussion with a physician

Ο Other sources (Please, mention it)

**Have you had a discussion with your physician about rabies?**

Ο Yes Ο No

**Were there any awareness & vaccination campaigns held for rabies in your geographical area?**

Ο Yes Ο No

**Have you been bitten or attacked by an animal before?**

Ο Yes Ο No

**Do you know anyone who has been bitten or attacked by animals before (a family member, a friend, or another)?**

Ο Yes Ο No

**Do you know anyone diagnosed or died from rabies before (a family member, a friend, or another)?**

Ο Yes Ο No

**KNOWLEDGE ABOUT RABIES AND RABIES PROPHYLAXIS**

**Knowledge about Rabies**

**Rabies is:**

- Viral disease
- Bacterial disease
- Parasitic disease

**Animals that transmit rabies:** “You can choose more than one answer.”

- Dogs
- Cats
- Rats
- Bats
- Horses
- Donkeys
- Cows And Buffalos
- Wolves
- Goats And Sheep

**Mode of transmission:** “You can choose more than one answer.”

- Biting
- Scratches
- Air
- Contaminated foods and drinks
- The saliva of an injured animal on an open wound.

**Signs of rabies in animals are:** “You can choose more than one answer”

- Changes in animal behavior.
- Self-mutilation
- Depraved appetite.
- Chasing people and a tendency to bite
- Bark continuously
- Sensitivity to light
- Animals have trouble moving and even paralysis.
- Unable to drink and eat.
- Excessive saliva
- Violent seizure

**Signs of rabies in human beings are:** “You can choose more than one answer”

- Flu-like symptoms (fever, headache, discomfort)
- Tingling and itching sensation at the site of the bite
- Weakness in the affected limb
- Hyperactivity and excitable behavior
- Convulsion
- Hallucination and delirium
- Difficulty swallowing
- Excessive salivation
- Fear of water
- Paralysis or partial paralysis
- Coma

**Do you think a man attacked by a rabid animal can recover?**

Ο No, I don’t think so.

Ο Yes with treatment

Ο Yes without treatment

**Can rabies be treated after the onset of clinical signs?**

Ο Yes Ο No

**Knowledge about Rabies prophylaxis**

**What would you do if you underwent an animal attack?** “You can choose more than one answer.”

- Wash the wound thoroughly with soap and water.
- Apply an antibiotic cream or ointment and cover the bite.
- Apply a disinfectant such as alcohol, betadine, or Dettol.
- Apply pressure with a bandage if bleeding.
- Leave wound without care.
- Go to the hospital for treatment.
- Other procedures (please, mention it)

**Do you think there is a vaccine for rabies?**

Ο Yes Ο No

**When rabies post-exposure prophylaxis should be taken?**

- Within the first 24 hours of the time of being bitten.
- After more than 24 hours of biting exposure time.
- After 1-2 weeks

**Who should take the pre-exposure prophylaxis of rabies?** “You can choose more than one answer”

- Farmers or people who live in rural areas.
- Wildlife workers
- Animal control officers
- People who have pets at home
- People who live in context with stray dogs and cats.
- People who work with rabies virus in labs
- Travelers to rabies-endemic area

**ATTITUDE**

Rabies is a viral disease that affects the nervous system of mammals, including humans. It is caused by the rabies virus, which is typically transmitted through the saliva of an infected animal, usually through a bite or scratch. The virus infects the central nervous system, causing inflammation in the brain and spinal cord. Once symptoms appear in the infected human, the disease is almost always fatal, so it is important to seek medical attention immediately if you have been bitten by an animal that may be infected with the virus.

Rabies is a preventable disease, and there are vaccines available for both humans and animals. If you live in an area where rabies is common, it is important to take precautions to avoid exposure to infected animals, such as keeping pets vaccinated and avoiding contact with wildlife.

**Do you think that rabies is a risk to human health?**

Ο Yes Ο No

**On a scale of 10, how do you rate the level of danger from rabies? (As 1 = very mild and 10 = very severe)**

**Do you think that rabies is a fatal disease?**

Ο Yes Ο No

**On a scale of 10, how do you support undergoing rabies post-exposure prophylaxis? (As 1 = strongly disagree and 10 = strongly agree)**

**On a scale of 10, how do you recommend undergoing rabies pre-exposure prophylaxis for the vulnerable group? (As 1 = strongly disagree and 10 = strongly agree)**

**Do you think that injecting a rabies vaccine as soon as possible after being bitten by suspected rabid animals is a must?**

Ο Yes Ο No

**Do you think that completing the full courses of vaccination after being bitten by a suspected rabid animal is a must?**

Ο Yes Ο No

**BARRIERS OF RABIES PROPHYLAXIS**

In this section, we are trying to explore potential barriers that prevent people from undergoing rabies prophylaxis.

**Each of the following may be a factor preventing you or other people from undergoing prophylaxis. For each of them, please indicate if it is a factor for you (Y/N).**

Ο Lack of knowledge about rabies vaccine

Ο Lack of knowledge about rabies

Ο the hospital in your area doesn’t have the required facilities for rabies prophylaxis.

Ο Fear of the process

Ο Financial Burden

Ο Previous bad experience (allergy, local abscess…)

Ο Feeling uncomfortable about dealing with healthcare staff.

**Among the previously mentioned factors (listed below), what is the most important factor that would prevent you or other people from undergoing rabies prophylaxis?**

Ο Lack of knowledge about vaccine

Ο Lack of knowledge about rabies

Ο The hospital in your area doesn’t have the required facilities for rabies prophylaxis

Ο Fear of the process

Ο Financial Burden

Ο Previous bad experience

Ο Feeling uncomfortable about dealing with healthcare staff

**PRACTICE OF RABIES PROPHYLAXIS**

**Post-exposure prophylaxis? (This section is only applied to participants who have undergone animal attack)**

**Did you undergo an attack or be bitten by an animal?**

Ο Yes Ο No

**Did you take the rabies post-exposure vaccine from the nearest hospital?**

Ο Yes Ο No

**When did you start your first dose?**

- Within the first 24 hours of the time of being bitten
- After more than 24 hours of biting exposure time
- After 1-2 weeks

**Did you complete the full vaccine schedule (total 5 doses)?**

Ο Yes Ο No

**Rabies Pre-exposure Prophylaxis (This section is only applied to vulnerable groups)**

**Are you one of the vulnerable populations for rabies?**

- Veterinarians.
- Farmers or people who live in rural areas.
- Wildlife workers
- Animal control officers
- People who have pets at home
- People who live in context with stray dogs and cats.
- People who work with rabies virus in labs
- Travelers to rabies-endemic area

**Did you think it’s a must to take the pre-exposure prophylaxis?**

Ο Yes Ο No

**Did you take the rabies pre-exposure vaccine from the nearest hospital?**

Ο Yes Ο No

**Did you complete the vaccine schedule (total 3 doses)?**

Ο Yes Ο No
